# Double burden of malnutrition amongst patients with first-episode schizophrenia in a psychiatric hospital: A 1-year follow-up study

**DOI:** 10.4102/sajpsychiatry.v26i0.1564

**Published:** 2020-11-10

**Authors:** Justus U. Onu, Portia N. Osuji

**Affiliations:** 1 Department of Mental Health, Faculty of Medicine, Nnamdi Azikiwe University, Awka, Nigeria; 2 Department of Neurosurgery, University College Hospital, Ibadan, Nigeria

**Keywords:** burden, double, malnutrition, first-episode, schizophrenia

## Abstract

**Background:**

Despite the burgeoning data on the double burden of malnutrition (DBM) in sub-Saharan Africa, longitudinal studies to examine malnutrition amongst first-episode schizophrenia are uncommon in the modern literature.

**Aim:**

To determine the extent of nutritional variations amongst persons with schizophrenia at intervals of 1-year treatment follow-up.

**Setting:**

This study was conducted at the Federal Neuropsychiatric Hospital, Enugu, Nigeria.

**Methods:**

Consecutive incident cases that fulfilled the criteria for schizophrenia were recruited into the study. After a baseline assessment, 206 incident cases of schizophrenia were followed up at 4th, 8th, 12th weeks, 6 months and 1 year for indicators of nutritional outcome. The body mass index (BMI) was used to measure the nutritional status amongst the study participants. Changes in the BMI across intervals of follow-up were examined using repeated measures analysis of variance, whereas the socio-demographic and clinical variables were evaluated as predictors of outcome using multiple regression analysis.

**Results:**

After 1 year of treatment with antipsychotics, the prevalence of underweight decreased from 19.9% (95% CI, 19.8% – 20.0%) at baseline to 16.0% (95% CI, 15.9% – 16.1%) at 1 year, but the prevalence of overweight/obesity increased from 29.1% (95% CI, 29.0% – 29.2%) at baseline to 43.2% (95% CI, 43.0% – 43.3%) at 1 year of follow-up. The predictors of BMI at 1 year were antipsychotic medication (32.7% variance), duration of vagrancy (24.0%) and age at onset (20.0%).

**Conclusion:**

The finding of coexistence of undernutrition and overnutrition across the intervals of treatment follow-up underscores the need for comprehensive interventions to address both extremes of malnutrition amongst patients with schizophrenia.

## Introduction

The concept of double burden of malnutrition (DBM) first emerged in 1992 and was defined as the coexistence of both undernutrition and overnutrition or diet-related non-communicable diseases (NCDs) in the same individual, household or a population across the life course.^1^ Double malnutrition affects all countries and socio-economic class, and the drivers have been summarised in the various changes the world is experiencing, namely nutrition, demographic and epidemiological transition.^1^ In sub-Saharan Africa, DBM poses a real and growing health challenge with high levels of undernutrition and a rising burden of overweight and obesity and diet-related NCDs.^2^

Schizophrenia is a chronic disorder affecting about 1% of the population with heterogeneous treatment outcome.^3^ There are reports of widening mortality gap amongst patients with schizophrenia in comparison with the general population.^4^ The higher risk of mortality amongst this population has been explained by various factors, including nutrition.^5^ Nutrition is recognised as a key determinant of well-being and a contributor to human capital development.^2^ There is growing evidence that nutrition may be of central importance to the manifestation of schizophrenia.^6,7^ The exact nature of the relationship between nutrition and various facets of the disease is not clear.^8^ Various authors suggest a complex dynamic relationship in relation to cause, effect and prognostic importance.^6,7,8,9^

Historically, famine and starvation were the common causes of malnutrition and have remained so till date. A growing concern is the malnutrition related to diseases, especially chronic physical and mental disorders.^8,10,11^ Psychiatric patients are known to have increased risk of malnutrition,^12,13,14^ yet physical examinations and nutritional assessments are not routinely performed and followed up by psychiatrists and other mental health professionals.^15^ Although literature on the DBM amongst patients with schizophrenia is sparse globally,^16^ Sugai et al.^13^ reported the prevalence of underweight and overweight + obesity amongst patients with schizophrenia in a Japanese psychiatric hospital as 13.8% and 30.2%, respectively. Although most previous studies have centred their findings on the indices of overnutrition (i.e. overweight and obesity) and metabolic disturbances amongst patients with schizophrenia;^5,17^ emerging evidence shows that underweight (i.e. undernutrition) is increasingly being recognised as a health risk which can lead to increased mortality.^18^ A recent meta-analysis of studies that specifically mentioned underweight amongst patients with schizophrenia identified only 17 studies globally with majority (23.5%) of them amongst Japanese in-patients.^16^ This study reported a pooled prevalence of underweight of 6.2%.^16^ However, when these studies were dichotomised between Japanese and non-Japanese populations, the pooled prevalence was 17.6% and 4.6%, respectively.^16^

In response to the malady of malnutrition in the population, the United Nations Decade of Action on Nutrition and Sustainable Development Goals’ double-duty actions has simultaneously and synergistically addressed undernutrition and overweight, obesity and diet-related NCDs more especially in the sub-Saharan Africa.^1^ Despite this, there is a paucity of data on the burden of double malnutrition amongst patients with schizophrenia receiving treatment in Nigeria. In Europe and North America, patients with schizophrenia treated with antipsychotic medications have a high prevalence of obesity and metabolic syndrome.^5^ However, in Africa, the burden of undernutrition remains huge with co-existing overnutrition.^2^ In the light of these issues, the following research questions became pertinent to nutritional research amongst patients with schizophrenia in Africa:

- What proportions of patients with schizophrenia have different levels of nutritional status (i.e. underweight, normal and overnutrition (defined in the methodology section) at each interval of 1-year treatment follow-up?
- What are the socio-demographic, clinical and psychosocial (including social support) predictors of nutritional outcome at 1-year of treatment follow-up?

## Materials and methods

### Study design and population

The details of the study methodology have been published elsewhere.^19^ However, this was a longitudinal naturalistic treatment follow-up study, carried out amongst the incident cases of schizophrenia for various nutritional outcomes. Consecutive incident cases of schizophrenia seen in the study centre were recruited into the study and followed up for 1 year. Patients aged 18–60 years were included in the study. Patients with schizophrenia of suspected organic aetiology or with medical or psychiatric comorbidities were excluded. As this was a naturalistic and observational study, the researcher did not attempt to interfere with the treatment measures put in place by the managing team. However, the treatments and investigations were documented.

### Diagnostic interview

Diagnosis of schizophrenia was made using the ICD-10 criteria for schizophrenia and confirmed using the Mini International Neuropsychiatric Interview (MINI). First, using the screening sections of the modules of the MINI, the researcher sought to screen out the presence of comorbid major mental disorders. A detailed medical history with full physical examination (including neurological examination) was conducted to exclude the presence of comorbid physical conditions.

Thereafter, the socio-demographic questionnaire was administered; this questionnaire contained items to assess socio-demographic characteristics and other nutritional variables (e.g. food refusal, changes in appetite and eating of non-nutritious materials).

The body mass index (BMI) or the Quetelet Index, which is a measure of weight in kilogram divided by height in metre squared, was used to determine the nutritional status across the intervals of treatment follow-up. The BMI has been known as a reliable measure of body proportions and composition, thinness or undernutrition.^20^ In 1992, a task force of the International Dietary Energy Consultative Group subcommittee on nutrition suggested that BMI should be used to define adult chronic dietary deficiency.^21^ The great advantage of this index is the ease of use, the low cost, the good correlation with the fat mass and the association with morbidity and mortality.^22^ In this study, nutritional status was classified using the cut-off of BMI score proposed by the World Health Organization as follows: underweight (BMI score less than 18.5 kg/m^2^), normal (BMI score 18.5–24.9 kg/m^2^), overweight (BMI ≥ 25 kg/m^2^) and obesity (BMI ≥ 30 kg/m^2^).^23^ However, undernutrition was the term used for underweight, whereas overnutrition was used for the categories of overweight and obesity.

The baseline assessments were completed at the emergency and crisis intervention unit or within 1 week of admission for admitted participants. The baseline weight and height were assessed, and BMI was calculated using the BMI formula (i.e. weight (in kg)/height squared (in meter). In addition, clinical severity of symptoms were rated using the Positive and Negative Symptoms Scale (PANSS),^24^ whereas the social support was assessed using the Multi-dimensional Scale of Perceived Social Support (MSPSS).^25^ This was applied when the patient was judged to have become clinically stable and able to complete the questionnaire. The Social Support Scale was either given to the patient or read aloud for the patient to indicate the answer as it applied to him or her.

For follow-up, participants were assessed at 4th, 8th, 12th weeks, 6 months and 1 year after the baseline. Participants were assessed using all the variables as described for the baseline assessment. Participants who missed their appointment were traced using phone numbers, family contact and next of kin’s address or phone number. A record of participants lost to follow-up was made.

### Data analysis

The statistical analysis was performed using the Statistical Package for Social Sciences, version 20 (IBM-SPSS 20). Data were entered and analysed based on the last observation carried forward (LOCF). Repeated measures analysis of variance was used to assess significant differences in change in BMI scores across the period of follow-up. The relationship between BMI at baseline, age at onset, duration of vagrancy, clinical severity and social support was assessed using Spearman’s correlation analysis. Multivariate analyses using step-wise multiple regressions were used to assess the predictors of nutritional outcome (BMI at 1 year follow-up). All tests of significance were two-tailed at the 5% level of significance and confidence interval estimation of 95%.

### Ethical consideration

Approval for this study was obtained from the Ethics and Research Committee of the Federal Neuropsychiatric Hospital, Enugu, Enugu State, Nigeria, with reference number FNHE/HTR/REA/VOL.11/356. This was approved on 15 August 2017 as part of a larger study on nutritional outcome of patients with schizophrenia. International ethical norms and standards were strictly adhered to at all times. Verbal and written consent were obtained from all the participants. Participation was voluntary.

## Results

Table 1 shows the socio-demographic characteristics of the 206 schizophrenia participants. The study participants were mostly young (mean age 26 years), females (58.7%), single (63.6%), had high school education (56.0%) and employed (57.8%). Table 2 shows the prevalence of underweight at baseline, 4th, 8th, 12th week, 6 months and 1 year (95% CI) were 19.9% (19.8% – 20.0%), 17.0% (16.9% –17.1%), 16.5% (16.4% – 16.7%), 16.0% (15.9% – 16.1%), 16.0% (15.9% – 16.1%) and 16.0% (15.9% – 16.1%), respectively. The prevalence of overnutrition (overweight + obesity) at baseline, 4th, 8th, 12th week, 6 months and 1 year (95% CI) were 29.1% (29.0% – 29.2%), 36.9% (36.8% – 37.0%), 39.3% (39.2% – 39.4%), 40.3% (40.2% – 40.5%), 41.7% (41.6% – 41.8%) and 43.2% (43.0% – 43.3%), respectively.

**TABLE 1:** Socio-demographic and clinical characteristics of the study participants, *N* = 206.

Socio-demographic variables	*N*	%	Mean ± s.d./Median (IQR)
Age (years)	-	-	26.16 ± 6.84
Age at onset (years)	-	-	26.33 ± 6.15
Dosage of antipsychotics (chlorpromazine equivalent in milligram)	-	-	500.00 (450.00)
Duration of illness (in months)	-	-	6.00 (22.00)
**Gender**			
Male	85	41.3	-
Female	121	58.7	-
**Marital status**			
Living with a partner	56	27.2	-
Not living with a partner	150	72.8	-
**Educational status**			
≤6 years	65	31.6	-
>6 years	141	68.4	-
**Occupational status**			
Employed	119	57.8	-
Unemployed	87	42.2	-
**Living circumstance**			
Alone	26	12.6	-
With family member	135	65.5	-
Homeless (vagrant)	45	21.9	-
**Medications**			
First generation	130	63.1	-
Second generation	72	35.0	-
Combined (typical and atypical)	4	1.9	
**Medication compliance**			
Regular	99	48.1	-
Irregular	107	51.9	-

IQR = interquartile range; s.d., standard deviation.

**TABLE 2:** Nutritional status across the intervals of treatment follow-up using last observation carried forward and real attrition data.

Time	Nutritional status	Using LOCF data		Time	Nutritional status	Real data (considering attrition)	
		*n*	%			*n*	%
Baseline (*n* = 206)	Underweight	41	19.9	Baseline (*n* = 206)	Underweight	41	19.9
	Normal	105	51.0		Normal	105	51.0
	Overweight and obesity	60	29.1		Overweight and obesity	60	29.1
4th week (*n* = 206)	Underweight	35	17.0	4th week (*n* = 179)	Underweight	26	14.5
	Normal	95	46.1		Normal	105	58.7
	Overweight and obesity	76	36.9		Overweight and obesity	48	26.8
8th week (*n* = 206)	Underweight	34	16.5	8th week (*n* = 126)	Underweight	16	12.7
	Normal	91	44.2		Normal	75	59.5
	Overweight and obesity	81	39.3		Overweight and obesity	81	27.8
12th week (*n* = 206)	Underweight	33	16.0	12th week (*n* = 114)	Underweight	5	4.4
	Normal	90	43.7		Normal	63	55.3
	Overweight and obesity	83	40.3		Overweight and obesity	46	40.4
6 month (*n* = 206)	Underweight	33	16.0	6 month (*n* = 113)	Underweight	4	3.5
	Normal	87	42.3		Normal	58	51.3
	Overweight and obesity	86	41.7		Overweight and obesity	51	45.1
1 year (*n* = 206)	Underweight	33	16.0	1 year (*n* = 112)	Underweight	3	2.7
	Normal	84	40.8		Normal	50	44.6
	Overweight and obesity	89	43.2		Overweight and obesity	59	52.7

LOCF, last observation carried forward.

Note: Undernutrition (i.e. Underweight) = BMI < 18.5, Normal = BMI ≥ 18.5–24.9, Overnutrition (Overweight + obesity) = BMI > 25; the prevalence of underweight using LOCF data at baseline, 4th, 8th, 12th week, 6 months and 1 year (95% CI) were 19.9% (19.8% – 20.0%), 17.0% (16.9% – 17.1%), 16.5% (16.4% – 16.7%), 16.0% (15.9% – 16.1%), 16.0% (15.9% – 16.1%) and 16.0% (15.9% – 16.1%), respectively, whereas the prevalence of overnutrition (overweight + obesity) at baseline, 4th, 8th, 12th week, 6 months and 1 year (95% CI) were 29.1% (29.0% – 29.2%), 36.9% (36.8% – 37.0%), 39.3% (39.2% – 39.4%), 40.3% (40.2% – 40.5%), 41.7% (41.6% – 41.8%) and 43.2% (43.0% – 43.3%), respectively.

**FIGURE 1:**
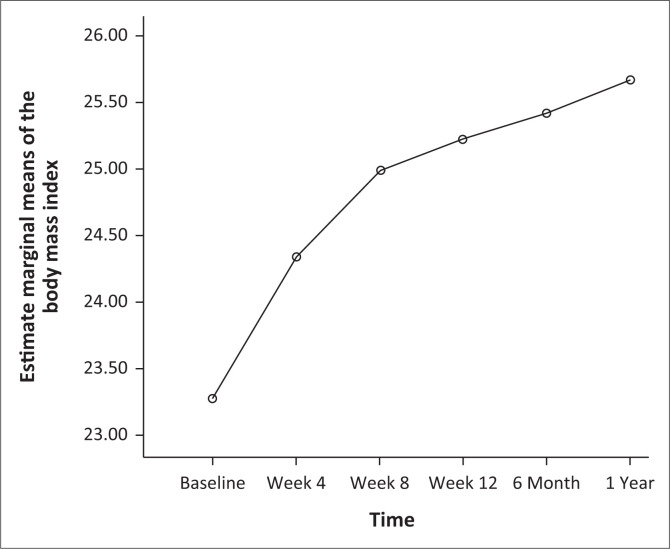
Change in the body mass index across the intervals of follow-up.

Table 3 shows a progressive increase in weight across the intervals of treatment follow-up and 80.6% of the participants had greater than 5% increase in weight at 1 year follow-up. Similarly, there was a significant increase in the BMI across the intervals of follow-up (*F* = 49.65; *p* < 0.001; partial eta squared = 0.3). Pairwise comparison shows that the significant difference was across most of the intervals (*p* < 0.001), and the exceptions were between 8th week and 12th week (*p* = 0.25) and between 12th week and 6 months (*p* = 0.41) as shown in Figure 1.

**TABLE 3:** Description of weight changes across the intervals of follow-up.

Time	Mean (s.d.) in kg		95% CI	< 5% increase in weight		≥ 5% increase in weight	
	*n*	%		*n*	%	*n*	%
Baseline	62.9	12.9	60.9–65.1	-	-	-	-
Week 4	65.7	13.2	63.4–68.0	47	22.8	159	77.2
Week 8	67.4	13.6	64.9–69.9	43	20.9	163	79.1
Week 12	68.1	13.7	65.6–70.6	44	21.4	162	78.6
6 months	68.6	14.4	65.9–71.3	44	22.4	162	78.6
1 year	69.5	14.6	66.9–72.1	40	19.4	166	80.6

CI, confidence interval; s.d., standard deviation.

The age at onset, duration of vagrancy, baseline BMI and antipsychotic medications were significant predictors of the BMI at 1 year, and they contributed 20.0%, 24.0%, 25.3% and 32.7%, respectively, of the variance as shown in Table 4.

**TABLE 4:** Relationship between body mass index at 1 year and body mass index at baseline, duration of vagrancy, age at onset, social support and disease severity.

Variables	*ρ*_s_ (*p*-value)		*R*^2^
	*n*	%	
BMI at baseline versus BMI at 1-year	0.8	< 0.001	0.253
Age at onset versus BMI at 1-year	0.3	0.02	0.200
Duration of vagrancy versus BMI at 1 year	−0.4	0.001	0.240
Social support versus BMI at 1 year	−0.1	0.54	-
Positive symptoms dimension score versus BMI at 1 year	−0.1	0.17	-
Negative symptoms dimension score versus BMI at 1 year	−0.1	0.76	-
General psychopathology dimension score versus BMI at 1 year	−0.2	0.02	-
Medications (antipsychotics)	-	-	0.327

*R*^2^, Coefficient of multiple determination; body mass index at 1-year is the dependent variable for the multivariate linear regression analysis.

## Discussion

The study was aimed at a naturalistic treatment follow-up of 206 incident cases of schizophrenia over 1-year period, with a view to highlight the burden of double malnutrition and to examine the potential predictors of nutritional status at 1 year.

This study is amongst the few African studies to examine the DBM amongst patients with schizophrenia using incident cases in a longitudinal design. The major strengths of the study are as follows: First, incident cases, predominantly neuroleptic naïve at the baseline were used to overcome the methodological problems inherent in prevalent and/or chronic cases. Second, they were followed-up for relatively a long period of 1 year using a well-validated measure of nutritional status.

The main findings of this study were as follows: (1) the DBM was evident across the intervals of treatment follow-up. The prevalence of undernutrition (i.e. underweight) and overnutrition (i.e. overweight + obesity) amongst the study participants were at baseline (19.9% vs. 29.1%), 4th week (17.0% vs. 36.9%), 8th week (16.5% vs. 39.3%), 12th week (16.0% vs. 40.3%), 6 months (16.0% vs. 41.7%) and 1 year (16.0% vs. 43.2%), respectively. (2) There was a progressive increase in weight and BMI across the intervals of treatment follow-up and (3) the age at onset, duration of vagrancy, being on antipsychotic medication and baseline BMI were predictors of nutritional outcome at 1 year.

The finding of the baseline prevalence of underweight and overweight + obesity of 19.9% and 29.1%, respectively, is consistent with previous reports.^13,14^ Assefa et al.^14^ in a cross-sectional study of patients with psychiatric disorders in an Ethiopian psychiatric hospital reported a prevalence of 20.0% and 23.4% for underweight and overweight + obesity, respectively. These studies share some similarities in the sense that the prevalence of overnutrition was higher than that of undernutrition in both studies. Additionally, both inpatient and outpatient were considered, and nutritional status was assessed using similar measure (i.e. the BMI). However, there are some methodological differences. For example, first-episode cases were used in this study unlike prevalent cases used in the Ethiopian study. More so, this study used a longitudinal design as against the cross-sectional design of the former. In addition, only patients with schizophrenia were included in this study, whereas the Ethiopian study used a heterogeneous group of people with mental disorders. This shows that this study is more likely to give a clearer picture of the nutritional status of patients with schizophrenia. Similarly, the finding of the higher prevalence of overnutrition when compared with undernutrition in this study is consistent with other international studies.^13,26^ Amongst Japanese patients with schizophrenia, Sugai et al.^13^ reported a prevalence of 13.8% and 30.2%, respectively, for undernutrition (i.e. underweight) and overnutrition (overweight + obesity). The lower prevalence of underweight of 13.8% amongst patients with schizophrenia in Japan when compared with 19.9% in this study may be explained by biological and sociocultural factors.^27^ Indeed, some authors have shown a higher prevalence of thin body build amongst Asian populations, and it is recommended that a lower BMI cut-off point should be used for Asian populations.^28^ Furthermore, the Japanese study was done amongst inpatients who are more likely to have severe disease. Interestingly, the prevalence of underweight and overweight + obesity across the intervals of treatment of follow-up was higher than the previously reported prevalence of underweight (2.0%) and overweight + obesity (26%) amongst community population in Nigeria.^29^ There are plausible explanations of the higher prevalence of underweight amongst patients with schizophrenia in this study when compared with that reported amongst the community population of Nigerians. First, the symptoms of the disease (e.g. anhedonia, loss of appetite, amotivation and easy fatigability) may limit food intake and lead to undernutrition. Similarly, positive symptoms of excitement may increase energy expenditure leading to a state of increased catabolism. Second, psychosocial difficulties such as poor social support, loss of employment and other indices of social disadvantage common amongst this population may limit the capacity of affected individual to have access to optimal nutrition. Third, biological factors such as chronic inflammation (e.g. gluten sensitivity), which is known to be common amongst patients with schizophrenia, may cause malabsorption and lead to undernutrition.^6^ Similarly, the higher prevalence of overweight and obesity amongst patients with schizophrenia when compared with the previous community prevalence in Nigeria (29.1% vs. 26.0%) could be explained by genetic, lifestyle and medication factors.^5,17^ However, it is noteworthy to state that although the proportion of patients with underweight decreased with treatment and a subsequent rise in the proportion of overweight + obese patients, both states coexisted throughout the intervals of treatment follow-up.

Another important finding in this study is the progressive reduction in the prevalence of undernutrition with treatment. In other words, there was a progressive increase in weight and BMI across the intervals of treatment follow-up. This finding is consistent with the robust reports in the literature that with treatment, there is a progressive increase in weight and BMI.^5,17^ This increase in weight as highlighted earlier can be explained by lifestyle factors, medications and inherent biological factors. It is also possible that with improvement in symptoms, patients’ appetite may improve and indices of social advantage restored; these factors may interact to improve nutritional status.

Concerning the predictors of nutritional status at 1 year, this study found that the age at onset of disease, duration of vagrancy, baseline body mass and use of antipsychotic medications were significant predictors of 1-year BMI. Previous studies have demonstrated that mental symptoms were significant risk factors of malnutrition amongst patients with mental disorders.^19,30^ Contrary to these previous reports, this study demonstrated a weak negative non-significant relationship between dimensions of psychopathology and BMI at 1 year. Although a previous study identified depressive symptoms as a risk factor for undernutrition,^30^ this study did not explore the effect of depressive symptoms rather the dimensions of symptoms were assessed. However, similar to the findings of this study, Kim et al.^12^ reported that living status and baseline weight were significant predictors of malnutrition amongst community-dwelling patients with schizophrenia. Several reports in the literature have linked homelessness with nutritional status of both mentally ill and persons without mental illness.^31,32^ It is known that many homeless persons eat fewer meals per day, lack food more often and are more likely to have inadequate diets.^31,32^ Wiecha et al.^31^ and Halsu et al.^32^ reported that homelessness is an important factor associated with the quality of nutrition and nutritional status.

## Limitations

One of the limitations of this study was the use of institution-based samples (care seekers); although it saved cost and time and is the predominant methodology in the literature, a community sample would have been more representative. The second limitation was the use of semi-structured questionnaire to assess feeding and/or eating behaviours; a structured questionnaire would have been more appropriate. Third, this study did not examine other biochemical variables to add to its depth. Fourth, the study did not include a control group that would have compared with general population. Finally, the attrition rate in this study was 45.6%; although within the range of 30% – 70% previously reported in a meta-analysis of attrition rates in longitudinal studies,^33^ the characteristics of those lost to follow-up may have affected the findings of this study.

## Conclusion

The main finding of this study is that the number of underweight patients stayed fairly similar but overweight increased over 1-year period of treatment follow-up. The finding of the coexistence of undernutrition and overnutrition amongst patients with schizophrenia underscores the need for proper nutritional assessment and a comprehensive multidisciplinary nutritional intervention to tackle the two extremes of malnutrition in this patient population.
